# Avian Influenza Screening in Captive Wild Birds and Biosecurity Appraisal of Zoological Gardens in Southwestern Nigeria

**DOI:** 10.1155/vmi/3419266

**Published:** 2025-10-29

**Authors:** Ridwan Olamilekan Adesola, Adetolase Azizat Bakre, Bamidele Nyemike Ogunro, Oladipo Omotosho, Oluwaseun Adeolu Ogundijo, Clement Adebajo Meseko, Bitrus Inuwa, Abdulafees Hamzat, Luqman Adeola Balogun, Damilola John Gbore, Usman Opeyemi Olatunji, Delower Hossain, Babatunde Ibrahim Olowu, Valentine Chidalu Okeke, Tomiwa Adisa, Quadri Olanrewaju Raji, Sodiq Tolase, Abdulhakeem Binhambali

**Affiliations:** ^1^Department of Veterinary Medicine, Faculty of Veterinary Medicine, University of Ibadan, Ibadan, Oyo, Nigeria; ^2^Veterinary Teaching Hospital, University of Ibadan, Ibadan, Oyo, Nigeria; ^3^Department of Veterinary Public Health and Preventive Medicine, Faculty of Veterinary Medicine, University of Ibadan, Ibadan, Oyo, Nigeria; ^4^Infectious Disease and Transboundary Animal Diseases, National Veterinary Research Institute, Vom, Plateau, Nigeria; ^5^Department of Statistics, Faculty of Physical Sciences, Federal University of Agriculture, Abeokuta, Ogun, Nigeria; ^6^Department of Medicine and Public Health, Faculty of Animal Science and Veterinary Medicine, Sher-e-Bangla Agricultural University (SAU), Sher-e-Bangla Nagar, Dhaka 1207, Bangladesh; ^7^Department of Veterinary Medicine, College of Veterinary Medicine, Michael Okpara University of Agriculture, Umudike, Abia, Nigeria; ^8^Department of Pathobiology, College of Veterinary Medicine, Tuskegee University, Tuskegee, Alabama, USA

**Keywords:** avian influenza, biosecurity programs, captive wild birds, contingency planning, surveillance

## Abstract

Avian influenza (AI) is a severe respiratory disease affecting wild and domestic birds globally. There is currently no approved vaccine for AI control in Nigeria. Therefore, biosecurity measures remain the primary preventive strategy. However, there is limited information on the AI carrier status of captive wild birds and the implementation of biosecurity programs (BPs) in zoological gardens across the country. This study aimed to screen captive wild birds for AI and evaluate the BPs in selected zoological gardens in southwestern Nigeria. Using a cross-sectional approach, cloacal swabs and freshly deposited faecal samples (*n* = 149) were collected from captive wild birds in seven zoological gardens in southwestern Nigeria following an AI outbreak in 2022. The samples were screened for AI viruses using RT-qPCR, and BPs in 13 consenting zoological gardens were assessed using a structured questionnaire. The responses were scored, and the BPs were categorised as ‘Poor', ‘Fair', or ‘Good'. Descriptive and inferential statistical methods were used to analyse the data. All samples tested negative for AI viruses. Documented institutional biosecurity and disease outbreak contingency plans were available in 84.6% of the zoos. Most of the zoos demonstrated good BPs across key categories, including vehicle and animal movement control (100%), food and water supply management (92.3%), enclosure hygiene, ground maintenance and waste disposal (84.6%), pest control (76.9%), and quarantine and sick animal management (69.2%). These findings suggest that the captive wild birds in zoological gardens in southwestern Nigeria are unlikely to serve as AI reservoirs, and most of the zoos have robust BPs that contribute to preventing AI and other avian diseases.

## 1. Introduction

Influenza A viruses (IAVs) are zoonotic pathogens that pose a constant health threat to animals and humans worldwide [1]. These viruses have been responsible for the most severe viral disease epizootic of avian origin in the 21st century [1]. The influenza viruses belong to the *Orthomyxoviridae* family characterised by an 8-segmented ribonucleic acid genome. The genome encodes more than 11 proteins, which includes hemagglutinin (HA) and neuraminidase (NA) [2, 3]. There are currently 18 and 11 identified subtypes of HA (H1–H18) and NA (N1–N11), respectively [4]. However, viruses with identical HA and NA may differ significantly in their genetic makeup, which often affects their pathogenicity and host specificity [2, 5]. The IAVs infect a diverse species of animals, with the majority of viruses being prevalent and endemic gastrointestinal tract infections in wild avian populations. Avian influenza viruses (AIVs) are IAVs that predominantly infect birds but can also cross species barriers to infect other animals [1]. Based on the severity of clinical symptoms and mortality rates observed in experimentally infected chickens, AIVs are classified into two pathotypes based on intravenous pathogenicity index test: high-pathogenicity avian influenza (HPAI) and low-pathogenicity avian influenza (LPAI) [6].

**Figure 1:**
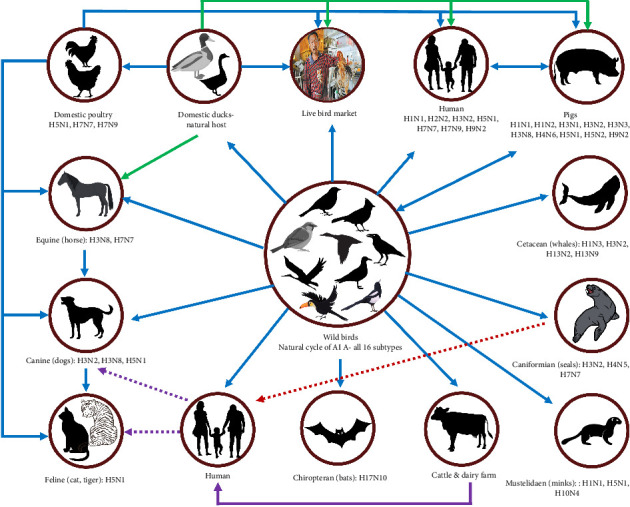
Transmission dynamics of avian influenza virus from wild birds to other hosts. The figure was adopted and modified from Kerstetter et al. [15].

HPAI is one of nine diseases listed under Section 10 (Aves) of the *Terrestrial Animal Health Code* by the World Organisation for Animal Health (WOAH) [7]. While primarily affecting birds, the disease occasionally breaches the species barrier, infecting humans and various domestic and wild animals, including dairy cows, horses, pigs, mink, seals, cats and dogs (Figure 1) [8, 9]. Since the first documented outbreak of HPAI in 1959, caused by the H5N1 serotype in poultry in Scotland [10, 11], numerous outbreaks have been recorded among bird populations. The disease was first transmitted to humans during an outbreak in Hong Kong in 1997 [12]. According to World Health Organization (WHO) reports, there were 878 cases of HPAI H5N1 infection in humans and 458 fatalities in 23 countries between 2003 and July 14, 2023 [13]. Nigeria recorded her first avian influenza (AI) outbreak in 2006, and there have been multiple AI outbreaks afterward [14]. There is compelling evidence associating wild birds with the ecology and epidemiology of HPAI in the sub-Saharan agroecological region [14].

Nigeria's zoological gardens (zoos) and wildlife parks are popular tourist destinations, particularly for children. They serve as dedicated exhibition spaces for a variety of wild animals, including birds. Despite the separation of wild captive bird species from visitors by mesh screens, zoo visitors are still at risk of contracting airborne pathogens such as AIV from captive birds because visitors usually stand close to the bird cages to get a nice view of the specimens. Since the first documented case of IA infection in a person in Nigeria, there have been concerns that a mutant strain of HPAI may spread from diseased captive wild birds in zoo settings to create a human epidemic within the country [16, 17].

Recently, there was an outbreak of avian influenza (AI) among birds in the aviary unit of one of the zoological gardens in southwestern Nigeria. The aetiology was confirmed by the National Veterinary Research Institute (NVRI) to be HPAI strain H5N1 (Unpublished). The outbreak resulted in a 100% mortality among the birds in the aviary unit of the zoo and was brought under control within 2 weeks. Although the outbreak has been curtailed, little is known about the current status of AI in other zoos in the region, or the biosecurity programs (BPs) implemented for disease prevention and control.

The term ‘biosecurity' refers to strategies and measures designed to prevent the spread of diseases from contaminated premises to neighbouring disease-free areas or to prevent the introduction of pathogens into disease-free premises [18]. Biosecurity can be implemented in two forms: biocontainment and bioexclusion [18]. Biocontainment involves restricting the virus within an infected unit, such as a hospital or farm, while bioexclusion focuses on preventing the introduction of the virus into a disease-free unit, depending on the specific context [18]. Implementing robust BPs remains the best method for preventing and controlling AIV in Nigeria because there is no certified AIV vaccine in the country. These measures must be implemented across the zoological gardens and wildlife parks in Nigeria to prevent the extinction of important wild bird species and preserve public health. There are numerous reports highlighting the importance of sound BPs in minimising the risk of introduction of AIV in captive wild birds [19–21]. Surveying the spread of AI in zoos in relation to existing BPs is important for building background knowledge to improve our understanding of the role of captive wild birds in the transmission of AI to other animals and humans, as well as to identify deficiencies in current BPs.

Therefore, this study aims (i) to screen for the presence of AIV among captive wild birds in major zoological gardens in southwestern Nigeria following recent AI outbreaks in the region and (ii) to assess the knowledge and attitudes of zoo personnel towards BPs for the prevention and control of AI and other diseases of public health and economic importance.

## 2. Materials and Methods

### 2.1. Study Sites

The study was carried out in 13 zoological gardens located in six states of southwestern Nigeria. Samples for AI screening were collected in the following seven consenting Zoological Gardens: University of Ibadan (UI Zoo) located Oyo State, Zoological Park, Federal University of Agriculture, Abeokuta (FUNAAB Zoo), and Olusegun Obasanjo Presidential Library Wildlife Park (OOPL Wildlife Park), located in Ogun State, Omu Resort Zoo and Greenfingers Wildlife Conservation located in Lagos State, Biological Garden, Obafemi Awolowo University (OAU Botanical Garden) located in Osun State, and Wildlife Park, Federal University of Technology, Akure (FUTA Wildlife Park) located in Ondo State while assessment of BPs was conducted in all 13 zoological gardens. The other six zoos are Agodi Gardens Zoo located in Oyo State, Q-brat Zoological Garden, Origin Zoo, and Lagos Zoo located in Lagos State, and Pretzels Animal Park located in Ogun State.

### 2.2. Ethics Statement

The ethical approval to carry out this study was obtained from the University of Ibadan Animal Care and Use Research Ethics Committee (UI-ACUREC/031-0423/13). Written permission was obtained from the directors of each of the zoological gardens. The consent of the participants was also obtained before the questionnaire administration.

### 2.3. Study Design for AI Surveillance

**Figure 2:**
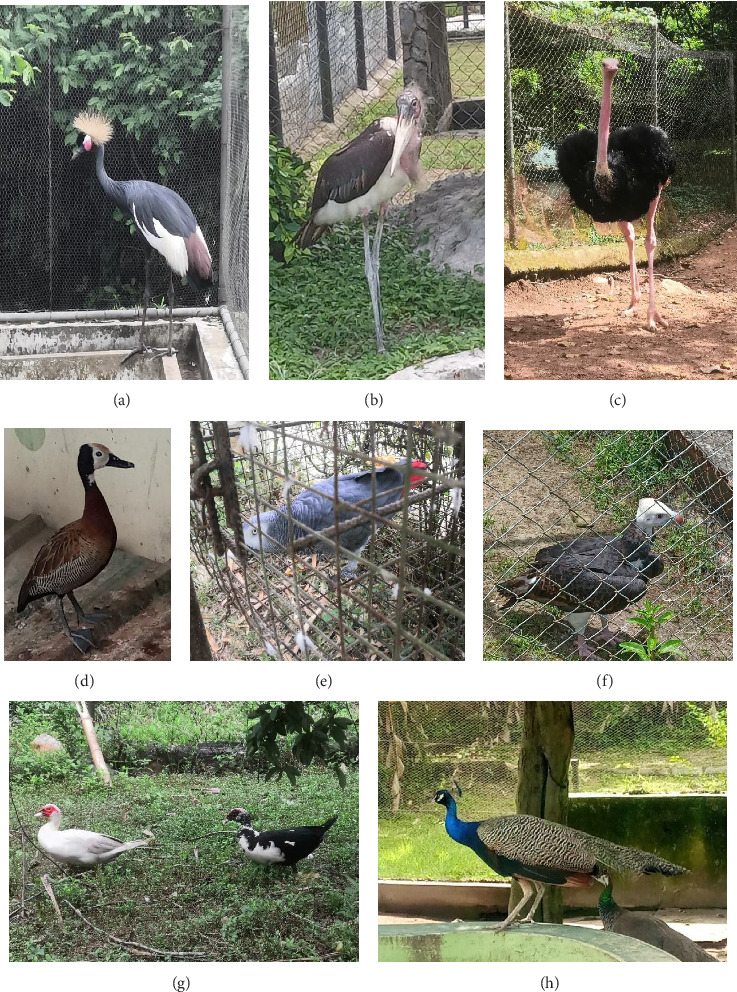
Pictorial representation of some of the sampled birds. (a) Black crowned crane (*Balearica pavonina*); (b) Marabou stork (*Leptoptilos crumenifer*); (c) Ostrich (*Struthio camelus*); (d) White-faced whistling duck (*Dendrocygna viduata*); (e) African grey parrot (*Psittacus erithacus*); (f) White-headed vulture (*Trigonoceps occipitalis*); (g) Muscovy ducks (*Cairina moschata*); (h) Indian Peafowl (*Pavo cristatus*)—male (blue colour) and female (green colour).

A cross-sectional approach was adopted for AI screening among the zoos using a stratified random sampling method. Samples for AI testing were collected from all apparently healthy captive wild birds at the seven zoological gardens between May, 21 and May 27, 2023. Some avian species were confined inside net cages, while others were allowed to roam freely within the natural habitat of the zoological park, usually with perimeter fencing (Figure 2). The birds were identified using a field guide [22].

### 2.4. Sample Collection and Processing

Cloacal swabs and freshly deposited faecal samples were collected from each bird and deposited in 1-mL glycerol-based virus transport medium and transported in an ice box to the Avian Diseases Unit, Department of Veterinary Medicine, University of Ibadan for sample sorting and storage (at −20°C). The samples were further transported to the Regional Support Laboratory for Avian Influenza and Newcastle Disease, National Veterinary Research Institute, Vom, Nigeria, for analysis using a standardised protocol as previously described [23].

### 2.5. Molecular Detection of AIV

The genomic viral RNA was extracted from the samples according to standard protocol [24]. Matrix (M) gene was amplified from the extracted RNA samples using quantitative reverse-transcriptase polymerase chain reaction (RT-qPCR) assays on a Rotor-Gene Q MDxmachine, model 5-plex (QIAGEN Co, CA, Germany). A 25-μL reaction mixture that contained 5 μL of RNA, 1.5 μL of each primer, M + 25 5′AGATGAGTCTTCTAACCGAGGTCG-3′ and M − 124 5′TGCAAAAACATCTTCAAGTCTCTCTG-3′ (5 μM), 2.5 μL of M + 64 FAM probe 5′ TCAGGCCCCCTCAAGCCGA-3′ (1 μM), 12.5 2X RT-PCR master mix, 0.2 μL of enzyme mix (Quantitect multiplex) and 1.8 μL of RNAse-free water was used under the following cycling conditions: The process involved 20 min at 50°C and 15 min at 95°C, then 40 cycles at 94°C for 45 s and 60°C for 45 s [25].

### 2.6. Appraisal of BPs

Assessment of the existing BPs in all the consenting zoological gardens (*n* = 13) was done from September 2023 to June 2024 using a standard questionnaire developed by Korhonen [26]. The questionnaire was printed in English and had four sections (A, B, C and D). Section A collected general information on the zoos, Section B collected responses about existing biosecurity protocols and preventive veterinary medicine practices in the zoos, Section C assessed the knowledge of avian influenza among zookeepers, zoo managers, zoo owners, or veterinarians in the zoos and Section D evaluated the biosecurity facilities and practices on the zoos. The tool was assessed for content clarity, validity, relevance to the study, ease of response, consistency and grammatical errors by three expert reviewers who are not co-authors of this paper. They were administered in an online survey (Google Forms) to the zookeepers, zoo managers, zoo owners and veterinarians who were authorised to represent the zoological gardens. The questionnaire is available using the link: https://docs.google.com/forms/d/10YpcMwVQQIIoZQ9Y1ETLqsP2qDCIyU8t1IoZnj6p088/viewform?edit_requested=true.

### 2.7. Data Analysis

The AI screening results were summarised using descriptive statistics on Microsoft Excel® platform, and the results were presented as frequencies and proportions. The responses in each of the four categories of the questionnaires were coded and analysed to score the animal welfare and record keeping practice (Section A), knowledge of avian influenza (Section C) and biosecurity protocols observed (Section B and D) in each location. Responses collected under Section D were given a score between 0 (absence of BPs) and 1 (full presence of BPs), where 1 and 0 are representations of ‘Yes' and ‘No', respectively. Each respondent under each question that picked ‘Yes' was allotted one point for each correct option, i.e., ‘Yes'. All the correct responses were summed up to make the respondents score under each category of biosecurity programs (cat–BPs). Aggregate scores of each respondent were ranked on an ordinal scale: ‘Poor', ‘Fair' and ‘Good' based on their score on each cat-BPs item category. Therefore, respondents who had a cumulative sum of BPs score of < 3 points were rated as having poor BPs, whereas respondents who scored between 3 and 4 points and those who scored > 5 points were deemed to have Fair and Good BPs, respectively. Finally, we used the Chi-square analysis to test the association between the qualitative variables of BPs. Variables with *p* < 0.05 were regarded as statistically significant. Statistical Package for the Social Sciences software (SPSS version 25 for Windows) was used for the data analysis.

## 3. Results

### 3.1. AI Surveillance

Based on the Zoo avian population and distribution of bird species, we obtained a total of 149 samples from UI Zoo (36), FUNAAB Zoo (20), OOPL Wildlife Park (31), Omu Resort Zoo (31), Greenfingers Wildlife Conversation (20), OAU Botanical Garden (3) and FUTA Wildlife Park (8).

The detailed distribution of captive wild bird species, International Union for Conservation of Nature (IUCN) status (https://www.iucnredlist.org/), molecular analysis and prevalence of AIV in major zoological gardens in southwestern Nigeria are presented in Table 1. According to the IUCN status classification, sampled captive wild birds in our study are classified least concern (*n* = 45), not available (for domesticated birds) (*n* = 7), vulnerable (*n* = 5), endangered (*n* = 4) and critically endangered (*n* = 3) species of captive wild birds. The qRT-PCR result for all the 149 samples tested showed no amplification for the M-gene from the cloacal swabs and faecal samples.

### 3.2. BPs Assessment

**Figure 3:**
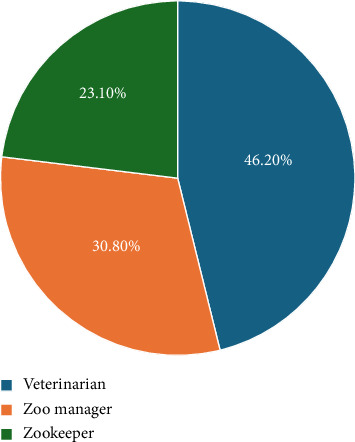
The roles of zoo representatives that provided responses for the assessment of biosecurity programs.

The 13 consenting zoological gardens (zoos) in the southwestern Nigeria region that participated in the BP assessment survey were represented by veterinarians (46.20%), zoo managers (30.80%) and zookeepers (23.10%) (Figure 3).

The zoos were in Lagos state (46.2%), Ogun state (23.1%), Oyo state (15.4%), Osun state (7.7%) and Ondo state (7.7%) (Table 2). Many of the zoos (38.5%) were owned by the federal government (Table 2). The total number of captive wildlife populations in most of the zoos (61.5%) was less than 100, and bird population was often (30.8%) between 20 and 29. Most zoos (38.5%) acquired between 5 and 9 birds annually but also lost at least one (84.6%) bird each year. Manual record keeping (53.8%) was the most prevalent mode of keeping animal records in zoos. The information recorded for each captive wild bird by the zoos includes bird species (100%), sex (92%), date of arrival at the aviary (85%), source of acquisition (77%), weight (62%), identification number (54%), date and place of birth (46%) and parent information (15%) (Table 2). About 76.9% of the surveyed zoos identified their animals with cage/house numbering.

All the zoos were aware of the possible infectious diseases that can afflict their aviary. The most listed diseases were AI (69%) and Newcastle disease (62%). While other less listed diseases were fowlpox (15%), histoplasmosis (8%), coccidiosis (8%), metabolic diseases (8%), infectious bursal disease (8%), avian respiratory disease (8%), mycoplasmosis (8%), salmonellosis (8%), chronic respiratory disease (8%), parrot tuberculosis (8%), infectious bronchitis (8%), colibacillosis (8%) and Marek's disease (8%) (Table 3). The pests most observed are rats (77%), houseflies (54%), lizards (46%) and free wild birds (38%) (Table 3).

About 84.6% of the zoos had instituted biosecurity plans and had documented disease outbreaks in the past (Table 3). As part of the biosecurity preventive measures, most zoos did not vaccinate nor knew the vaccines used to vaccinate their birds (46%) (Figure 3). Chlorine (38%), saponated cresol (31%), sodium hypochlorite (23%) and chloroxylenol (23%) are the most commonly used disinfectants in the zoo. Most of the zoos claimed to dispose of their wastes through waste bins (38%) and by burying (23%). Almost all zoos (92.3%) train their staff on the proper biosecurity measures (Table 3).

The awareness of AI among the zoos was very high (100%), and 92.3% claimed that it affects captive wild birds (Table 4). While only 46.2% had experienced its outbreak in the past and believed that there is no drug to treat AI. In addition, 76.9% had institutional plans of action to prevent avian influenza (Table 4).

The zoos practiced good BPs under vehicle and animal movement control (100%), food and water supply (92.3%), enclosure hygiene, ground maintenance and waste disposal (84.6%), pest control (76.9%) and quarantine and management of sick animals (69.2%) categories (Table 5). Food and water supply, enclosure hygiene and ground maintenance and waste disposal BPs were significantly (*p* < 0.05) part of the institutional-specific biosecurity plan practiced by the zoos to prevent and control AI (Table 5).

## 4. Discussion

In this study, we provide current information on avian influenza carrier status of captive wild birds and appraise the biosecurity architecture for the prevention of infectious diseases in some zoological gardens in southwestern Nigeria. Our investigation was prompted by an unpublished outbreak of a highly pathogenic AI that killed several exotic birds in one of the participating zoological gardens in 2022. We utilised highly sensitive molecular techniques for the surveillance of the virus in the apparently healthy captive wild bird population. Previous studies have investigated the prevalence and molecular characterisation of the AI virus among wild birds in some African countries including Cameroon [27], South Africa [28], Kenya [29] and Nigeria [17, 30–32], but information is lacking on the level of biosecurity in the affected facilities. However, biosecurity remains an important outbreak mitigation strategy, especially against AI.

Our surveillance revealed absence of AI in all the studied facilities, which implies that the virus is not circulating among the captive birds but may be introduced from free-ranging wild birds or from the introduction of new animals without proper quarantine procedures. Previous surveys of AI in Nigeria have yielded mixed results. Our findings are similar to that of Snoeck et al.'s [30] study, who also detected zero prevalence of AI in Amurum Forest reserve, Plateau State, while Daodu et al. [32] and Ameji et al. [31], who utilised serological techniques, recorded a prevalence of 10.4%, and 4.5% in captive wild birds respectively, while Snoeck et al. [30] were able to detect AI viral gene in wild birds with 6.8% prevalence from Dagona Wildlife Sanctuary, Yobe State. Elsewhere, Philippa et al. [33] reported a 1.6% seroprevalence of AI in a Dutch zoo, and Desvaux et al. [34] detected AIV in 7 captive wild birds out of 8 in Cambodia's Phnom Tamao Wildlife Rescue Centre with RT-PCR. Serological techniques such as ELISA, as used in some of the studies, have the ability to detect both past and current infections and, hence, present a higher prevalence result than molecular techniques, which are designed to detect only current infections. According to Das et al. [35], the biggest risk with any diagnostic method is the possibility of false-negative results. With RT-PCR, false-negative may occur due to low virus titres, RT-PCR inhibitors, mismatched primers, poor RNA extraction, errors in setting up a reaction, and degradation of target RNA before amplification [36]. In our study, we instituted extra precautions to prevent false-negative results by running the test isolates with in-house positive and negative controls.

As avian influenza is a rapidly spreading viral disease, implementing strict biosecurity in zoological gardens and wildlife parks is not negotiable. An unpublished outbreak of highly pathogenic AI in 2022 was attributed to a breach in biosecurity procedures. The outbreak followed a mixing of a recently acquired batch of wild birds with already caged birds without going through adequate quarantine and screening. This error led to the loss of more than half of its captive wild bird population. However, prompt biocontainment measures were instituted to prevent the spread of AI at the facility. The 2022 AI outbreak in the facility is a reminder of the consequences of biosecurity breaches. Other factors that could influence the prevalence of AI in wild birds include factors such as the season, species, location and age of birds, as reported in wild birds in Europe and America [37–39].

In our assessment of the existing BPs in the 13 consenting zoological gardens in southwestern Nigeria, we observed that most of them were represented by veterinarians (46.2%), and the highest number of zoos (46.2%) were in Lagos state. This is because of the thriving tourism industry in Lagos state due to the large population of residents and tourists. Many of the zoos were owned by the federal government (38.5%) and educational institutions (30.8%). This is likely due to the capital-intensive nature of the establishment and maintenance of a zoo. Apart from being a tourism centre, the zoos also serve as training centres for students of the institutions, especially the veterinary medical, wildlife and ecotourism students. Another interesting finding is that the total number of captive wild animals in most zoos was less than 100, with birds constituting 20%–49% of the population. There was also a higher number of bird procurements than other species in the zoos within a year. This relatively high population of avian species may be due to their lower cost of procurement and maintenance and the fact that captive birds are known to attract tourists because of their ornamental nature [40].

Furthermore, we observed most zoos (53.8%) used a manual format of record keeping, and they recorded the birds' information such as bird species (100%), sex (92%), date of arrival at the aviary (85%), source of acquisition (77%), weight (62%), identification number (54%), date and place of birth (46%) and parent information (15%). This contrasts with Korhonen [26], who reported the extensive use of electronic form of recording keeping in Tallinn Zoo. Nigeria is classified as a low-middle-income country with limited advancement in technology compared to Estonia. The discrepancy between our findings and Korhonen [26] study might be a result of differences in the economic income and technological advancement in the study countries. The means of bird identification included cage or house numbering (76.9%). This is a regular identification practice in Nigeria to prevent any anatomical damage to the animals.

The most common diseases that were viewed as a threat to the wild bird population were AI (69%) and Newcastle disease (62%). These two diseases continue to rank high among disease threats to a wide range of domestic and wild avian species in Nigeria, with great economic and public health importance [41–43]. The good knowledge of AI and Newcastle disease displayed by the participants is related to the high prevalence of these diseases in avian species in Nigeria. Bala et al. [44] also reported a good knowledge of Newcastle disease among poultry farmers (100%) in Nasarawa. Free-range wild animals have been reported to be carriers of diseases and can also act as predators for captive wild animals in zoos [45]; we observed that rats are the most common pest (77%) in the zoos. In Nigeria, rats have been reported to be a reservoir of infectious pathogens such as Lassa fever virus [46], *Salmonella* sp. [47], Orthopox viruses [48], *Leptospira interrogans* [49], and endoparasites and ectoparasites [50]. Adequate control of rats is important to prevent the spread of these diseases to captive wild animals and humans working and visiting zoos.

Another notable finding from our study was that all the participants had a high awareness of AI (100%). We recorded a higher level of awareness of AI compared to Aiyedun et al. [51], who reported 47% awareness among zoo workers in three zoological gardens in another study area in Nigeria. The differences in the level of awareness between the studies may be due to the differences in the study area or the time of the study, as the 2022 AI outbreak in a prominent zoological garden in Nigeria must have heightened awareness among others. Also, Aiyedun et al. [51] focused on zoonotic diseases in zoological gardens; the participants might have limited knowledge about ‘zoonoses' and have impacted their responses, as some may not be sure whether AI is zoonotic or not. Regarding the prevention and control of AI, 76.9% of the participants had documented plans to prevent AI, 46.2% professed no treatment for AI and 46% did not administer any drug during outbreaks. As it is established, prevention is always better than cure; therefore, zoos in Nigeria need adequate plans for prevention and control AI to keep their wild birds and humans healthy. Depopulation, culling infected birds, disinfection and decontamination of farm equipment are some of the current control measures practiced in Nigeria for avian disease control [43]. Fasina et al. [52] recommend the combination of these measures with vaccination, for a cheaper and more effective control. There are no approved vaccines or effective treatments for AI in Nigeria. However, less than half of the participants knew this, which highlights the need for increased awareness about the prevention and control of AI among Nigerian zoos.

We found that 84.6% of the zoos had biosecurity institutional plans and documented action plans for disease outbreaks. Our findings are similar to those of Korhonen [26], who documented the existence of various biosecurity institutional plans practiced in Tallinn Zoo. Biosecurity is an essential practice needed in enclosed settings such as zoological gardens, but it may be difficult to implement, especially in low- and middle-income countries where funding may be a challenge and policies may be bypassed. The containment and spread of diseases in zoological gardens are dependent on the availability of good biosecurity plans. Despite the Newcastle disease endemicity in Nigeria, only 31% of zoos vaccinated their birds against the disease. A low level of awareness of vaccines used to vaccinate against Newcastle disease had also earlier been reported by Ibrahim et al. [53]. His study reported that 13% of poultry farmers were aware of and vaccinated their birds against Newcastle. In addition, we reported that 23% of the zoos did not vaccinate and did not know the vaccines required for the birds, while 15% of the zoos vaccinated their birds against AI. Vaccination is one of the important biosecurity measures that need to be practiced for every vaccine-preventable disease in zoos. However, there is low awareness of vaccination among the surveyed zoos, necessitating the need to revisit and implement awareness programs among the zoos. Since there is no approved AI vaccine in Nigeria, the use of the influenza vaccine by those zoos was against the national policy on poultry disease control, which needs to be stressed during the awareness program. One of the challenges that may arise with the use of unapproved vaccines is the possibility of mutation and evolution of new deadly strains of the pathogen, which may cause great harm to the captive wild birds and poultry industry. Studies are needed to evaluate the impacts of unapproved vaccines imported into Nigeria on the evolution of novel AIV strains.

Considering our assessment of BPs in the zoological gardens under the categories: (1) vehicle and animal movement control, (2) food and water supply, (3) enclosure hygiene, ground maintenance and waste disposal, (4) pest control, (5) work and hygiene procedures, (6) quarantine and management of sick animals, we observed that categories 1 (100%), 2 (92.3%), 3 (84.6%), 4 (69.2%) and 6 (76.9%) had a high level of good biosecurity practice in the zoos. Categories 2, 3 and 4 of the BPs were significantly (*p* < 0.05) part of the institutional-specific biosecurity plan practiced by the zoos to prevent and control AI. While categories 5 and 6 were not statistically significant and can be linked to one of the limited BPs associated with the cause and spread of the initial outbreak of AI in one of the zoos surveyed. It is known that work and hygiene procedures are very important in preventing the entry and spread of pathogens into zoos. Procedures include regular changes of staff clothes, footwear hygiene, handwashing, use of gloves and footbaths or mats. Some of these procedures were lacking in the surveyed zoos, and this contributed to the low scores in work and hygiene procedures in BPs. The high cost of some of these procedures might have caused the zoos not to have them. There is a need to increase financial support to zoos in Nigeria so they can invest in costly BPs. The impact of outbreaks will be costlier if not prevented. Another key BP that needs great attention is quarantine and management of sick animals. Quarantine of new animals is key to preventing the introduction of new diseases into the collection. It involves isolating new animals to monitor their health status for at least the first 30 days of acquisition. In our study, we perceived good quarantine practices among the zoos, but they were not statistically significant in preventing the outbreak of AI. This might be because some of the practices were not currently done appropriately in the zoos and need more attention. Among the practices that were not done properly under quarantine category are functional quarantine facilities, all-in/all-out principle, controlled/monitored facility entrances and dedicated quarantine equipment. To strengthen BPs in zoos in Nigeria, the establishment of functional quarantine facilities is important, as there will always be a need for replacement stock or addition to an existing collection. The requirements for the quarantine of each animal species vary, but for captive birds, a medium metallic cage can be used to quarantine them to examine their baseline health conditions, treat and administer vaccines [54]. The quarantine facilities must be strictly controlled, the equipment used in the quarantine facilities should not leave the premises, and the workers should thoroughly clean themselves after leaving the quarantine facilities. Lastly, the quarantine facilities should follow the all-in/all-out principle, which means that all animals in the quarantine facilities leave simultaneously [54]. The period should be restarted if there is a need to add a new animal during ongoing quarantine. Increasing awareness and implementing these BPs will heavily assist in preventing AI outbreaks among captive wild birds in Nigeria.

### 4.1. Limitation

One of the major limitations we encountered during this study was sample collection. There was a prior agreement between investigators and the zoo manager to only carry out noninvasive sample collection techniques, which led to the collection of the faecal samples and cloacal swab samples, rather than other or in addition to methods, such as oropharyngeal swabs, tracheal swabs or blood, which are the best samples to show AI prevalence in nonreservoir hosts. The zero prevalence recorded in the study might be a result of the sampling techniques used. However, this study advocates for good humans, animals and environmental health as it revealed the prevalence of AI in captive wild through enteric and environmental samples and the role of proper BPs in preventing and controlling AI in zoos.

## 5. Conclusion

We report zero prevalence of AI in captive wild birds in zoological gardens in southwestern Nigeria following the implementation of biocontainment measures after the 2022 Ibadan outbreak. Given the increasing global risk of HPAI genetic reassortment across species, consistent surveillance in zoos is essential to understand disease dynamics. Future studies should adopt a One Health approach, integrating serological and molecular methods to monitor birds, zoo workers and visitors. The conservation of wildlife remains critical, as these species both sustain ecosystems and can act as reservoirs for zoonotic pathogens. Notably, many zoological gardens in the study area already maintain biosecurity action plans, underscoring the importance of proper biosafety practices in reducing AI risk. We recommend strengthening quarantine systems for newly introduced birds, improving hygiene protocols for staff and visitors, and enhancing awareness among zoo stakeholders. While vaccination remains prohibited for AI control in Nigeria, promoting other preventive strategies will be vital to safeguard both animal and public health.

## Figures and Tables

**Table 1:** Distribution of captive wild birds, IUCN status, molecular analysis, and prevalence of avian influenza virus in major zoological gardens in southwestern Nigeria.

Name of zoo	Common name	Zoological name	IUCN status	Number sampled	M gene + ve/number sampled	AI prevalence (%)
Zoological Garden, University of Ibadan	African grey parrot	*Psittacus erithacus*	Endangered	1	0/1	0.00
	Black crowned crane	*Balearica pavonina*	Vulnerable	2	0/2	0.00
	Emu	*Dromaius novaehollandiae*	Least concern	1	0/1	0.00
	Helmeted Guinea fowl	*Numida meleagris*	Least concern	3	0/3	0.00
	Hooded vulture	*Necrosyrtes monachus*	Critically endangered	1	0/1	0.00
	Mallard duck	*Anas platyrhynchos*	Least concern	4	0/4	0.00
	Marabou stork	*Leptoptilos crumenifer*	Least concern	2	0/4	0.00
	Muscovy duck	*Cairina moschata*	Least concern	2	0/2	0.00
	Ostrich	*Struthio camelus*	Least concern	1	0/1	0.00
	Palm-nut vulture	*Gypohierax angolensis*	Least concern	2	0/2	0.00
	Indian peafowl	*Pavo cristatus*	Least concern	1	0/1	0.00
	Great white pelican	*Pelecanus onocrotalus*	Least concern	1	0/1	0.00
	Spur-winged goose	*Plectropterus gambensis*	Least concern	2	0/2	0.00
	White-faced whistling duck	*Dendrocygna viduata*	Least concern	6	0/6	0.00
	White goose	*Anser anser domesticus*	Not available	5	0/5	0.00
	White stork	*Ciconia ciconia*	Least concern	2	0/2	0.00

Zoology Park, FUNAAB	African grey parrot	*Psittacus erithacus*	Endangered	2	0/2	0.00
	Crowned crane	*Balearica pavonina*	Vulnerable	1	0/1	0.00
	Black kite	*Milvus migrans*	Least concern	1	0/1	0.00
	Mallard duck	*Anas platyrhynchos*	Least concern	5	0/5	0.00
	Ostrich	*Struthio camelus*	Least concern	2	0/2	0.00
	Indian peafowl	*Pavo cristatus*	Least concern	3	0/3	0.00
	White goose	*Anser anser domesticus*	Not available	6	0/6	0.00

Biological Garden, Obafemi Awolowo University.	Indian peafowl	*Pavo cristatus*	Least concern	1	0/1	0.00
	Ostrich	*Struthio camelus*	Least concern	1	0/1	0.00
	Black crowned crane	*Balearica pavonina*	Vulnerable	1	0/1	0.00

Wildlife Park, Federal University of Akure	African grey parrot	*Psittacus erithacus*	Endangered	1	0/1	0.00
	Mallard duck	*Anas platyrhynchos*	Least concern	3	0/3	0.00
	White goose	*Anser anser domesticus*	Not available	1	0/1	0.00
	Black crowned crane	*Balearica pavonina*	Vulnerable	1	0/1	0.00
	Ostrich	*Struthio camelus*	Least concern	2	0/2	0.00

Wildlife Park, Olusegun Obasanjo presidential library	Black crowned crane	*Balearica pavonina*	Vulnerable	1	0/1	0.00
	Greylag goose	*Anser anser*	Least concern	10	0/10	0.00
	Helmeted guineafowl	*Numida meleagris*	Least concern	5	0/5	0.00
	Hen	*Gallus gallus domesticus*	Least concern	2	0/2	0.00
	Mallard duck	*Anas platyrhynchos*	Least concern	1	0/1	0.00
	Muscovy duck	*Cairina moschata*	Least concern	1	0/1	0.00
	Ostrich	*Struthio camelus*	Least concern	2	0/2	0.00
	Indian peafowl	*Pavo cristatus*	Least concern	4	0/4	0.00
	Pekin duck	*Anas platyrhynchos domesticus*	Not available	1	0/1	0.00
	Spur-winged goose	*Plectropterus gambensis*	Least concern	2	0/2	0.00
	White-faced whistling duck	*Dendrocygna viduata*	Least concern	2	0/2	0.00

Omu Resort Park	Emu	*Dromaius novaehollandiae*	Least concern	1	0/1	0.00
	Golden eagle	*Aquila chrysaetos*	Least concern	1	0/1	0.00
	Hen	*Gallus gallus domesticus*	Least concern	1	0/1	0.00
	Mallard duck	*Anas platyrhynchos*	Least concern	5	0/5	0.00
	Marabou stork	*Leptoptilos crumenifer*	Least concern	1	0/1	0.00
	Ostrich	*Struthio camelus*	Least concern	2	0/2	0.00
	White-headed vulture	*Trigonoceps occipitalis*	Critically endangered	1	0/1	0.00
	Indian peafowl	*Pavo cristatus*	Least concern	2	0/2	0.00
	Feral pigeons	*Columba livia domestica*	Not available	11		0.00
	Lesser whistling duck	*Dendrocygna javanica*	Least concern	1	0/1	0.00
	White goose	*Anser anser domesticus*	Not available	5	0/5	0.00

Greenfingers Wildlife Conservation	African grey parrot	*Psittacus erithacus*	Endangered	4	0/4	0.00
	Emu	*Dromaius novaehollandiae*	Least concern	1	0/1	0.00
	Golden eagle	*Aquila chrysaetos*	Least concern	1	0/1	0.00
	Hen	*Gallus gallus domesticus*	Least concern	4	0/4	0.00
	Mallard duck	*Anas platyrhynchos*	Least concern	2	0/2	0.00
	Ostrich	*Struthio camelus*	Least concern	1	0/1	0.00
	Budgerigar	*Melopsittacus undulatus*	Least concern	1	0/1	0.00
	Indian peafowl	*Pavo cristatus*	Least concern	2	0/2	0.00
	Feral pigeons	*Columba livia domestica*	Not available	2	0/2	0.00
	Turkey	*Meleagris gallopavo*	Least concern	1	0/1	0.00
	Indian vulture	*Gyps indicus*	Critically endangered	1	0/1	0.00

Total				149		0.00

**Table 2:** Zoo information and record-keeping practice.

Variable		Frequency (%)
Location	Lagos	6 (46.2)
	Ogun	3 (23.1)
	Oyo	2 (15.4)
	Osun	1 (7.7)
	Ondo	1 (7.7)

Zoo owner	Federal government	5 (38.5)
	Educational institution	4 (30.8)
	State government	2 (15.4)
	Privately owned	2 (15.4)

Overall wildlife population	< 50	4 (30.8)
	50–100	4 (30.8)
	101–149	2 (15.4)
	150–199	2 (15.4)
	> 200	1 (7.7)

Bird population	10–19	2 (15.4)
	20–29	4 (30.8)
	30–39	3 (23.1)
	40–49	3 (23.1)
	Above 50	1 (7.7)

No birds procured (in the last 1 year)	0	2 (15.4)
	1–4	3 (23.1)
	5–9	5 (38.5)
	10–14	2 (15.4)
	15 and above	1 (7.7)

No birds hatched and survived (in the 1 year)	0	8 (61.5)
	1–4	3 (23.1)
	10–14	1 (7.7)
	15 and Above	1 (7.7)

Birds' mortalities (in the last 1 year)	0	2 (15.4)
	1–4	11 (84.6)

Format of keeping record	Manual	7 (53.8)
	Manual and electronic	6 (46.2)

Data recorded for each bird	Bird species	13 (100)
	Sex	12 (92)
	Date of arrival at the aviary	11 (85)
	Source of acquisition	10 (77)
	Weight	8 (62)
	Identification number	7 (54)
	Date and place of birth	6 (46)
	Parent information	2 (15)

Means of identifications	Cage or house numbering	10 (76.9)
	Natural body markings	2 (15.4)
	Body tags	1 (7.7)

Abbreviations: No = Number, Zoo = Zoological garden.

**Table 3:** Biosecurity and preventive veterinary medicine program available in Zoos.

Variable		Frequency (%)
Is the zoo aware of the possible infectious diseases that may threaten the aviary collection?	Yes	13 (100)
	No	0 (0)

List the infectious diseases that may threaten the aviary collection	Avian influenza	9 (69)
	Newcastle disease	8 (62)
	Fowl pox	2 (15)
	Histoplasmosis	1 (8)
	Coccidiosis	1 (8)
	Metabolic diseases	1 (8)
	Infectious bursal disease	1 (8)
	Avian respiratory disease	1 (8)
	Mycoplasmosis	1 (8)
	Salmonellosis	1 (8)
	Chronic respiratory disease	1 (8)
	Parrot tuberculosis	1 (8)
	Infectious bronchitis	1 (8)
	Colibacillosis	1 (8)
	Marek's disease	1 (8)

What pests are observed at the aviary?	Rats	10 (77)
	Houseflies	7 (54)
	Lizards	6 (46)
	Free wild birds	5 (38)
	Snakes	1 (8)
	None	1 (8)

Does the zoo have an institution-specific biosecurity plan?	Yes	11 (84.6)
	No	2 (15.4)

Does the zoo have a documented plan of action in case of disease outbreak?	Yes	11 (84.6)
	No	2 (15.4)

What vaccinations are administered? List	NDV LASOTA	4 (31)
	None	3 (23)
	Not known	3 (23)
	Influenza vaccine	2 (15)
	Paramyxovirus vaccine & Poxvirus vaccine	1 (8)

What disinfectants are used in the zoo? List	Chlorine	5 (38)
	Saponated cresol	4 (31)
	Sodium hypochlorite	3 (23)
	Chloroxylenol	3 (23)
	Hydrochloride and formalin	1 (8)
	Neo din	1 (8)
	Liquid soap	1 (8)
	Detergents	1 (8)

How are waste products disposed?	Waste bin	5 (38)
	Buried	3 (23)
	Burning	2 (15)
	Burning, buried & waste bin	1 (8)
	Buried or burning	1 (8)

Does the staff receive regular training in the relevant aspects of zoo biosecurity	Yes	12 (92.3)
	No	1 (7.7)

**Table 4:** Awareness of zoos on avian influenza.

Variable		Frequency (%)
Are you aware of avian influenza?	Yes	13 (100)
	No	0 (0)

Does the disease affect captive wild birds?	Yes	12 (92.3)
	No	1 (7.7)

Have you experienced an outbreak of avian influenza in the past?	Yes	6 (46.2)
	No	7 (53.8)

Does the zoo have a documented plan of action for the prevention of avian influenza	Yes	10 (76.9)
	No	3 (23.1)

Are there drugs for treatment of avian influenza?	Yes	4 (30.8)
	No	6 (46.2)
	Not sure	3 (23.1)

What drugs do you use to treat avian influenza? List	None	6 (46)
	Not sure	3 (23)
	Antiviral	3 (23)
	Antibiotics	1 (8)

Is there always a veterinarian available during avian influenza emergency outbreak?	Yes	12 (92.3)
	No	1 (7.7)

**Table 5:** Levels of biosecurity programs in surveyed zoos.

Categories	Biosecurity programs	Poor *n* (%)	Fair *n* (%)	Good *n* (%)	*χ* ^2^	*p* value
1	Vehicle and animal movement control			13 (100)		
2	Food and water supply	1 (7.7)		12 (92.3)	5.958	0.015^∗^
3	Enclosure hygiene, ground maintenance and waste disposal	1 (7.7)	1 (7.7)	11 (84.6)	6.017	0.049^∗^
4	Pest control		4 (30.8)	9 (69.2)	5.318	0.021^∗^
5	Work and hygiene procedures	1 (7.7)	6 (46.2)	6 (46.2)	0.197	0.906
6	Quarantine and management of sick animals	1 (7.7)	2 (15.4)	10 (76.9)	2.245	0.325

*Note:χ* ^2^: Chi-square test.

^∗^Statistically significant.

## Data Availability

The data that support the findings of this study are available from the corresponding author (delowervet@sau.edu.bd) and first author (adesolaridwanolamilekan@gmail.com) upon reasonable request.
